# Established Beta Amyloid Pathology Is Unaffected by TREM2 Elevation in Reactive Microglia in an Alzheimer’s Disease Mouse Model

**DOI:** 10.3390/molecules26092685

**Published:** 2021-05-04

**Authors:** Qiuju Yuan, Xiaodong Liu, Yi Zhang, Yan-Fang Xian, Juntao Zou, Xie Zhang, Pengyun Huang, You-Qiang Song, Zhi-Xiu Lin

**Affiliations:** 1 Faculty of Medicine, School of Chinese Medicine, The Chinese University of Hong Kong, Hong Kong; lisaxian@cuhk.edu.hk (Y.-F.X.); zoujuntao@163.com (J.Z.); zhangxie@link.cuhk.edu.hk (X.Z.); huangpengyun2012@163.com (P.H.); 2 Brain Research Centre, Faculty of Medicine, The Chinese University of Hong Kong, Hong Kong; 3 Department of Anaesthesia and Intensive Care, Faculty of Medicine, The Chinese University of Hong Kong, Shatin, Hong Kong; greg.xd.lau@gmail.com; 4 Li Ka Shing Faculty of Medicine, School of Biomedical Sciences, The University of Hong Kong, Pokfulam, Hong Kong; yi1zhang@yahoo.com (Y.Z.); songy@hku.hk (Y.-Q.S.)

**Keywords:** Alzheimer’s disease, TREM2, microglia activation, Aβ deposition, spinal cord

## Abstract

Several genetic studies have identified a rare variant of triggering receptor expressed on myeloid cells 2 (TREM2) as a risk factor for Alzheimer’s disease (AD). However, findings on the effects of TREM2 on Aβ deposition are quite inconsistent in animal studies, requiring further investigation. In this study, we investigated whether elevation of TREM2 mitigates Aβ pathology in TgCRND8 mice. We found that peripheral nerve injury resulted in a robust elevation of TREM2 exclusively in reactive microglia in the ipsilateral spinal cord of aged TgCRND8 mice at the age of 20 months. TREM2 expression appeared on day 1 post-injury and the upregulation was maintained for at least 28 days. Compared to the contralateral side, neither amyloid beta plaque load nor soluble Aβ40 and Aβ42 levels were attenuated upon TREM2 induction. We further showed direct evidence that TREM2 elevation in reactive microglia did not affect amyloid-β pathology in plaque-bearing TgCRND8 mice by applying anti-TREM2 neutralizing antibody to selectively block TREM2. Our results question the ability of TREM2 to ameliorate established Aβ pathology, discouraging future development of disease-modifying pharmacological treatments targeting TREM2 in the late stage of AD.

## 1. Introduction

Alzheimer’s disease (AD) is characterized by synaptic and neuronal degeneration, culminating in cognitive decline. One of its pathological hallmarks is senile plaques in the brain [1]. These plaques are caused by extracellular accumulation of Aβ [1]. Several pathogenic mechanisms have been assumed although the cause of neurodegeneration in AD remains unclear [1]. One of the mechanisms is the accumulation of Aβ. Aβ is a peptide produced by the cleavage of amyloid precursor protein (APP) expressed in neurons with high concentrations. Therefore, targeting the Aβ peptide has been the primary focus for therapeutic interventions in AD [2].

Another important pathological hallmark of AD is reactive gliosis in the vicinity of extracellular Aβ plaques [3]. As in many other neurodegenerative diseases, whether the AD associated reactive gliosis drives or delays AD progression is contentious and may depend on innate immune-related genes expressed by microglia. 

Recently, genome wide association studies using exome sequencing have resulted in the discovery of a series of rare mutations in triggering receptor expressed on myeloid cells 2 (TREM2) gene, including R47H, which may increase the risk of AD by three-fold [4,5,6]. TREM2 is mainly expressed by microglia in which TREM2 plays important roles in microglial phagocytosis and inflammatory responses [3,7]. Recent studies have shown that TREM2 might directly bind to Aβ peptides and hence participate in Aβ clearance [8,9]. It was therefore expected that TREM2 deficiency may lead to increased Aβ burden. However, the reports on Aβ plaque in AD mice with TREM2-deficiency are inconsistent. Different outcomes including augments [10,11], no effects [12,13], and even reductions of extracellular plaque pathology were reported in TREM2-deficient mice [14]. How Aβ is affected by TREM2 needs further studies. Although intensive studies have investigated the mechanism underlying the TREM2-plaque interaction, most of the studies are adopting TREM2 deletion. Few studies, if any, are conducted to assess the role of TREM2 elevation in Aβ pathology [15].

The transgenic TgCRND8 mouse is a widely used animal model of AD [16,17,18]. Our previous studies have suggested that Aβ deposition can be detected in the spinal cord dorsal horn of TgCRND8 mice [16,17,18]. A recent study found that spinal injury upregulated TREM2 expression in the microglia in spinal cord dorsal horn [19]. The present study aimed to investigate the association between TREM2 elevation and amyloid removal in TgCRND8 mice. Brachial plexus nerve ligation was adopted to induce the elevation of TREM2 in the reactive microglia. The roles of TREM2 expressing microglia in Aβ burden in spinal dorsal horn were then investigated. 

## 2. Results

### 2.1. Age-Dependent Amyloid Burden in the Spinal Cord of TgCRND8 Mice

**Figure 1:**
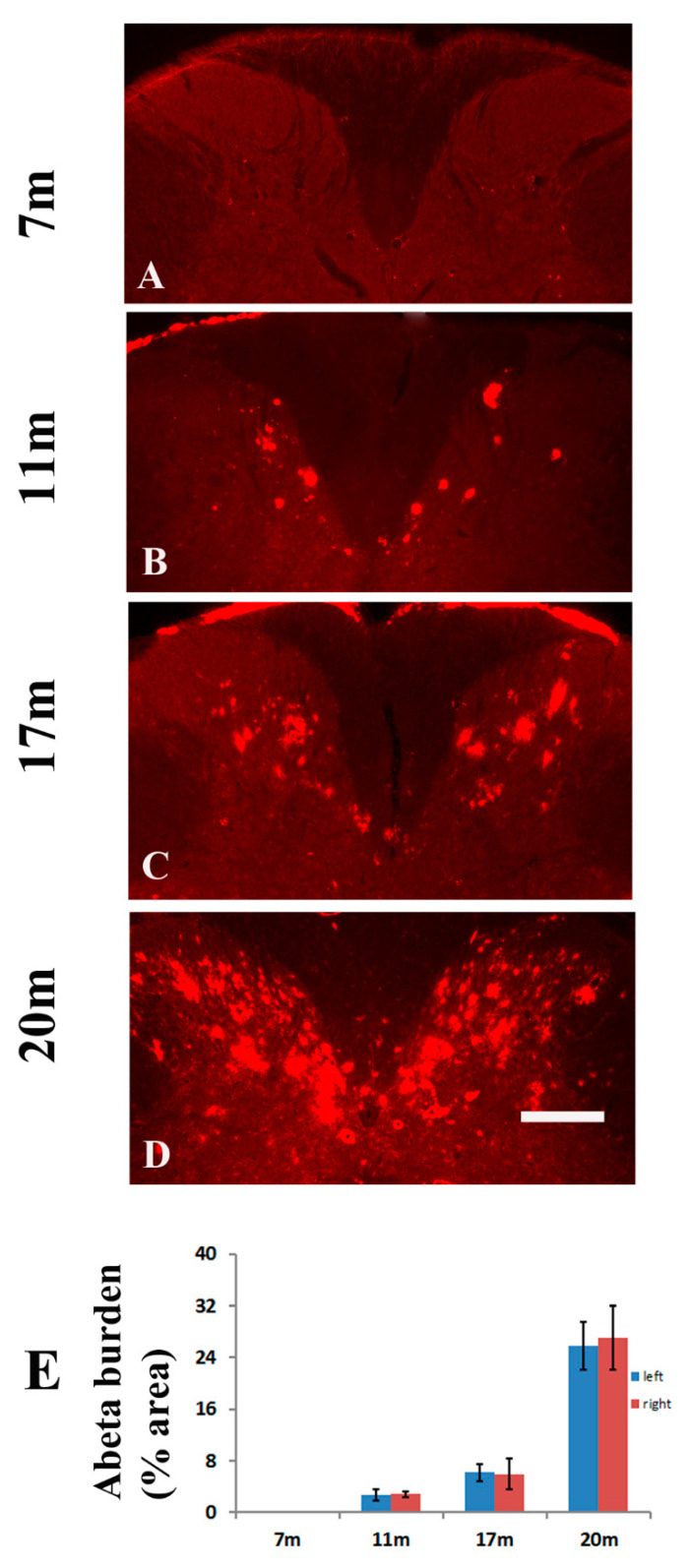
Age-dependent accumulation of Aβ plaques in the dorsal horn of the spinal cord of TgCRND8 mice. Representative photomicrographs of progressive Aβ pathology in cross sections of the cervical cord of TgCRND8 mice at 7, 11, 17, and 20 months of age. Aβ immunofluorescence study revealed an absence of Aβ pathology in the spinal cord at the age of 7 months (**A**). A few Aβ plaques could be observed in the dorsal horn at the age of 11 months (**B**) and increased with aging ((**C**,**D**), respectively). (**E**) Quantitative analysis of amyloid burden in the dorsal horn of the spinal cord of TgCRND8 mice. For each individual animal analyzed, amyloid burden in the left was compared with the burden in the right side in the dorsal horn. No difference in amyloid burden could be observed. Scale bar, 150 μm.

**Figure 2:**
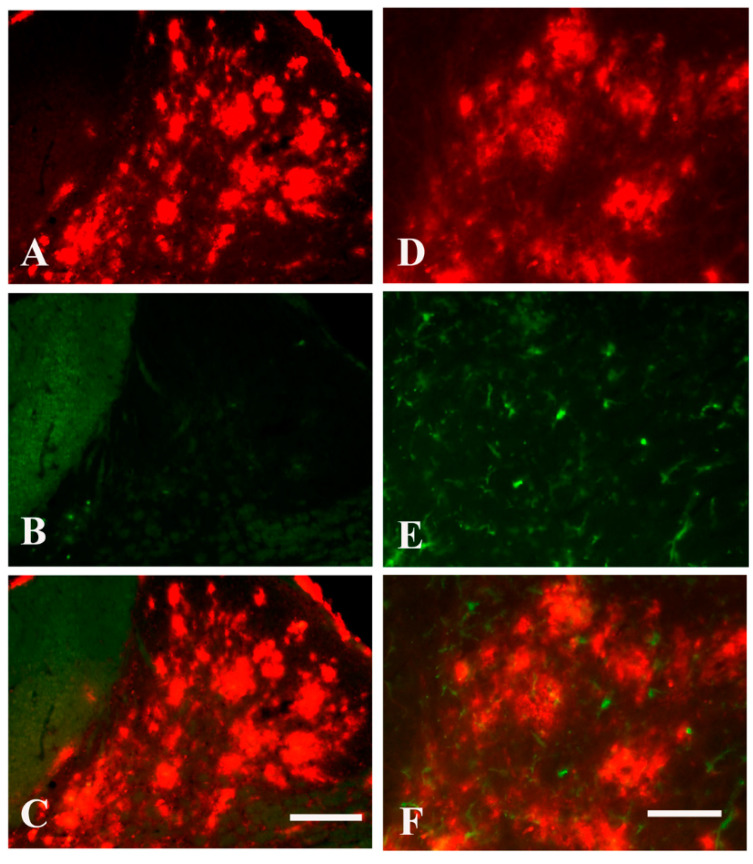
Characterization of Aβ plaques in the spinal cord of TgCRND8 mice at the age of 20 months. (**A**–**C**): Double labeling of Aβ (red)/thioflavin S (green) revealed the Aβ plaques are thioflavin S negative, indicating the plaques are diffuse plaques. (**D**–**F**): Double labeling of Aβ (red)/IBA-1 (green) revealed no activated microglia (green) in the immediate vicinity of Aβ plaques (red) in the spinal cord. Scale bar in (**C**), 100 μm. Scale bar in (**F**), 75 μm.

To investigate amyloid deposition and reactive microglia dependent clearance of Aβ in the spinal cord, TgCRND8 transgenic mice were applied [20]. Cervical cord sections were stained with human Aβ-specific antibody Bam-10 and visualized by indirect immunofluorescence. The representative images of mice aged 7 (Figure 1A), 11 (Figure 1B), 17 (Figure 1C), and 20 (Figure 1D) months were shown in Figure 1. As expected [16,17,18], amyloid deposition in the spinal cord increased with age. There was no significant difference in the Aβ burden between the left and right sides of the spinal cord (Figure 1E). To further assess Aβ plaques in the spinal cord of TgCRND8 mice, we performed double staining of Aβ/thioflavin S and Aβ/IBA-1 (a marker for microglia) in the spinal cord sections from TgCRND8 mice aged 20 months. Consistent with our previous studies [17], almost all Aβ plaques were not stained by thioflavin S staining (Figure 2A–C). Meanwhile, the double staining of Aβ/IBA-1 showed that no activated microglia (Figure 2D–F) were presented in the immediate vicinity of Aβ plaques (red) in the spinal cord (Figure 2D–F), suggesting that diffuse plaques were prominent in the spinal cord. 

### 2.2. Brachial Plexus Nerve Ligation Led to Microglia Activation in the Dorsal Horn of Spinal Cord in Tgcrnd8 Mice

**Figure 3:**
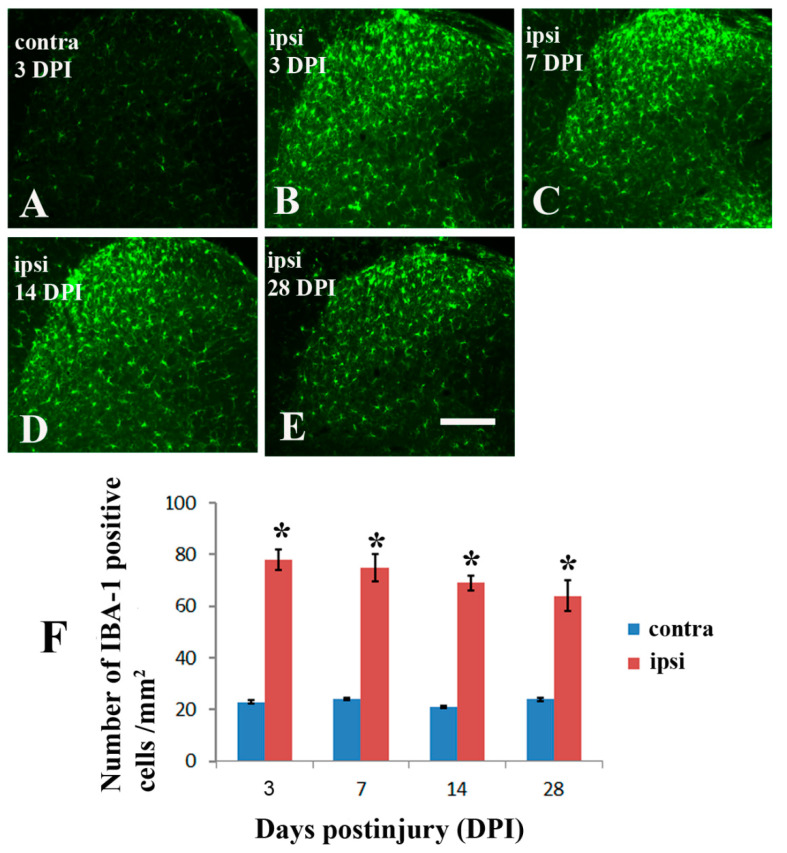
Immunohistochemistry for IBA-1 in spinal dorsal horns following nerve ligation of brachial plexus in TgCRND8 mice at the age of 20 months. (**A**): IBA-1 expression of the contralateral side (contra) following the nerve ligation. (**B**–**E**): IBA-1 expression of the ipsilateral side (ipsi) at 3, 7, 14, and 28 days after nerve ligation (DPI). (**F**): Comparison of changes in the IBA-1 expression at 3, 7, 14, and 28 days between the contralateral and ipsilateral side of dorsal horn. * Significant differences between the two sides, *p* < 0.01. Scale bar, 120 μm.

It has been well established that spinal nerve injury results in microglial activation in the dorsal horn of the spinal cord [21,22,23,24]. Thus, we hypothesized that brachial plexus nerve ligation might lead to microglia activation in the spinal cord dorsal horn of TgCRND8 mice aged 20 months. Compared with the contralateral side (Figure 3A), IBA-1 positive microglia was significantly increased in the ipsilateral dorsal horn as early as day 1 following nerve injury (Figure 3B). The injury-induced IBA-1 expression was continuously maintained in the ipsilateral dorsal horn on 7 (Figure 3C), 14 (Figure 3D), and 28 (Figure 3E) days postinjury. The quantitative analysis of IBA-1 expression at each time point was shown in Figure 3F. 

### 2.3. Brachial Plexus Nerve Ligation Induced TREM2 in Microglia in the Dorsal Horn of Spinal Cord in Tgcrnd8 Mice 

**Figure 4:**
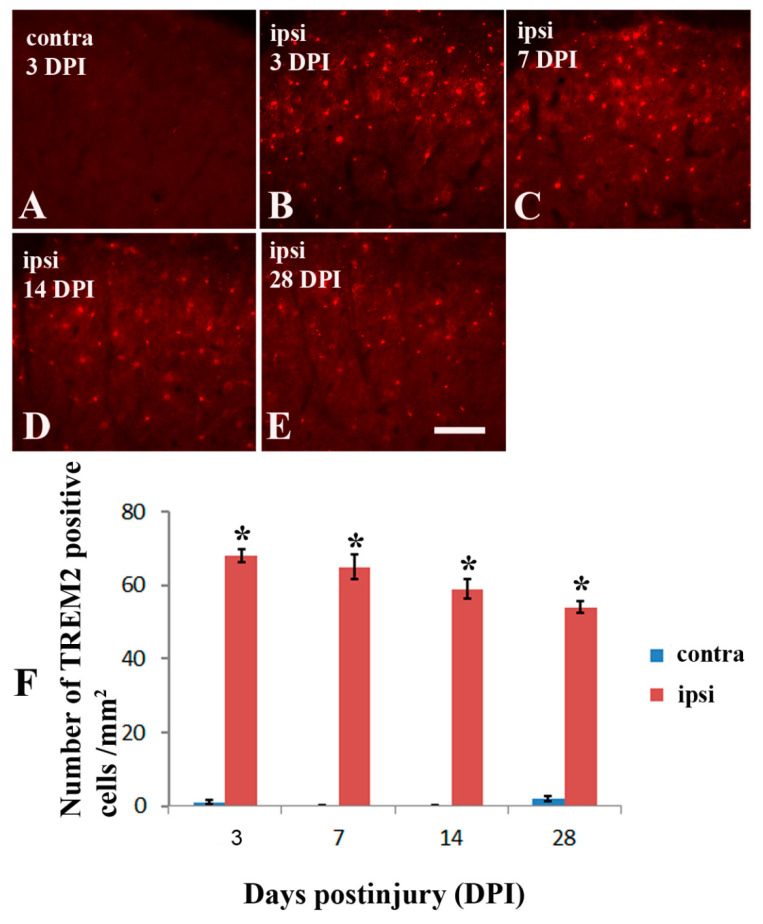
Immunohistochemistry for TREM2 in spinal dorsal horns following nerve ligation of brachial plexus in TgCRND8 mice at the age of 20 months. (**A**): TREM2 expression of the contralateral side (contra) following the nerve ligation. (**B**–**E**): TREM2 expression of the ipsilateral sides (ipsi) at 3, 7, 14, and 28 days after nerve ligation (DPI). (**F**): Comparison of changes in the TREM2 expression at 3, 7, 14, and 28 days between the contralateral and ipsilateral side of dorsal horn. * Significant differences between the two sides, *p* < 0.001. Scale bar, 75 μm.

**Figure 5:**
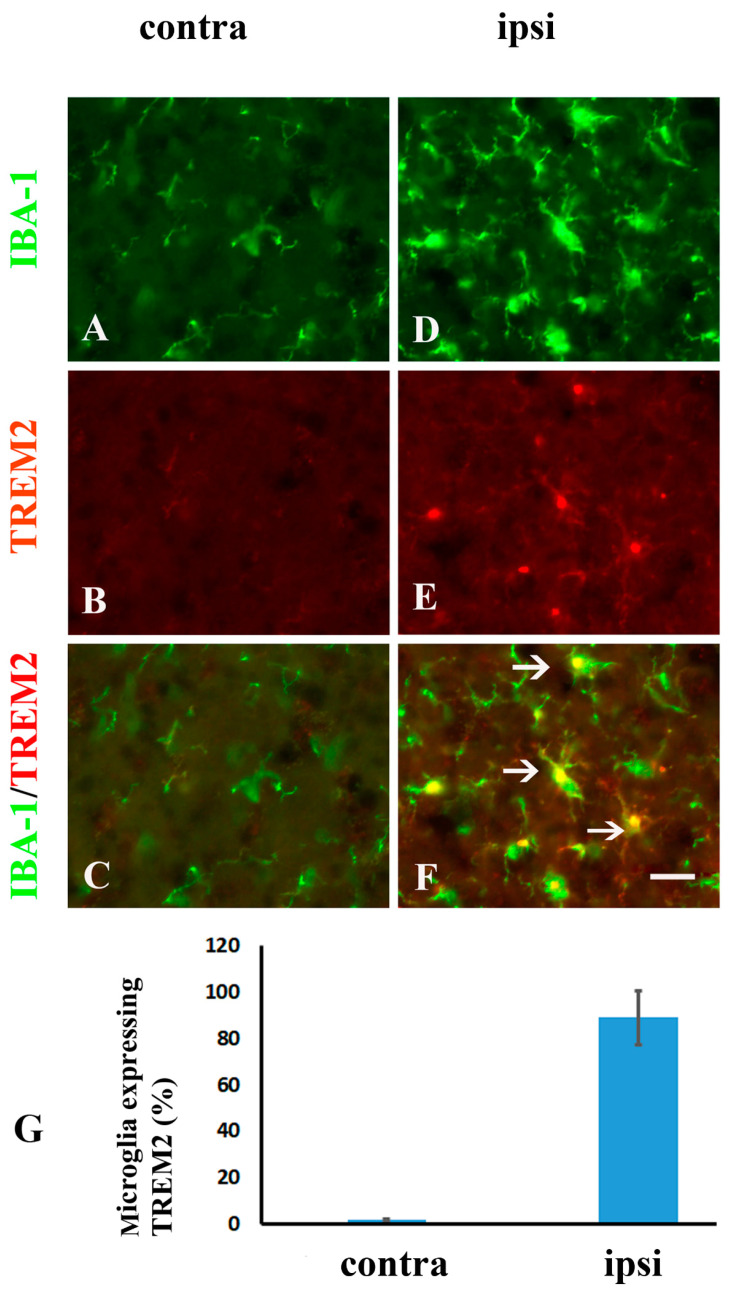
Double labeling of IBA-1(green)/TREM2 (red) in the contralateral (contra) (**A**–**C**) and ipsilateral (ipsi) (**D**–**F**) sides of the spinal cord of the TgCRND8 mice at 7 days postinjury reveals TREM2 is exclusively expressed in activated microglia following nerve ligation (arrows in (**F**)). (**G**): Quantitative analysis of microglia expressing TREM2 (%) in the contralateral (contra) and ipsilateral (ipsi) sides. Scale bar, 10 μm.

Consistent with the previous study that TREM2 was elevated in the spinal cord dorsal horn microglia following sciatic nerve injury [19], In the current model of brachial plexus ligation, TREM2 was also significantly increased in the ipsilateral dorsal horn from day 1 (Figure 4B,F) to day 7 (Figure 4C,F), day 14 (Figure 4D,F) and day 28 (Figure 4E,F), when compared with the contralateral side (Figure 4A,F). To identify the cell types expressing TREM2 after nerve injury, double immunostaining was performed using antibodies against TREM2 and IBA-1, a microglial marker. All TREM2 staining was colocalized with IBA-1-positive immunostaining (arrows in Figure 5F), suggesting that TREM2-expressing cells were microglia in the spinal cord dorsal horn (Figure 5D–F). 

### 2.4. Brachial Plexus Nerve Ligation Did Not Affect Amyloid Burden in Spinal Cord

**Figure 6:**
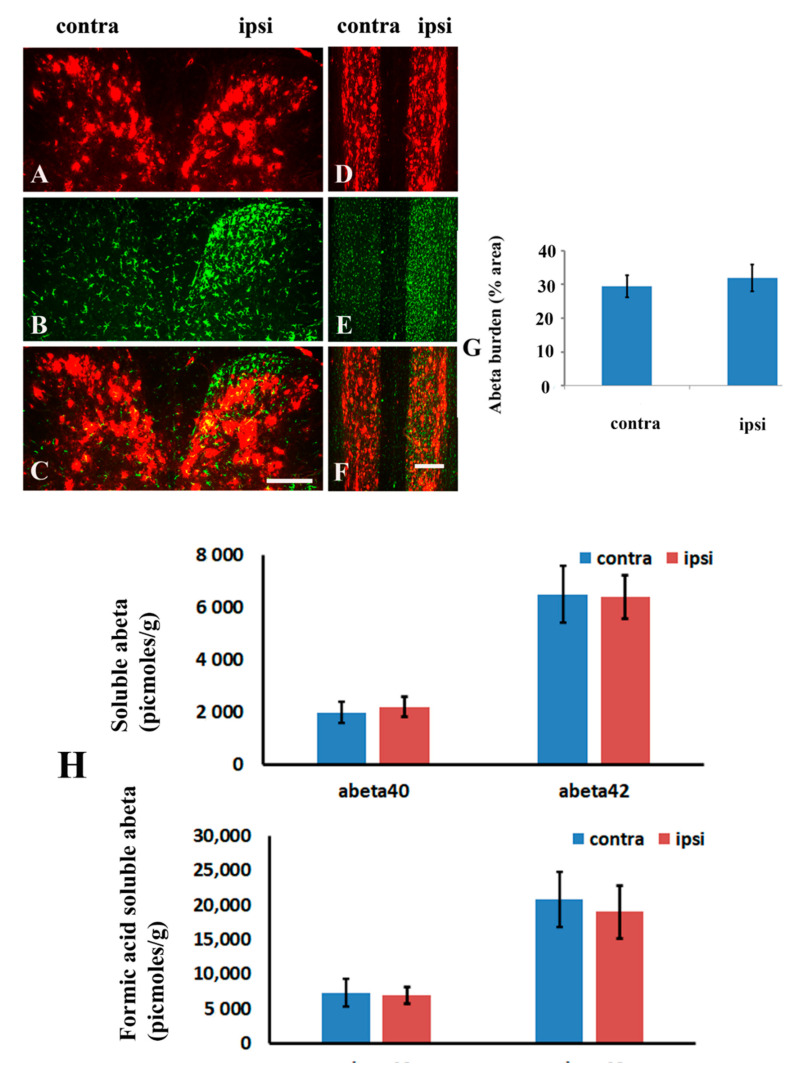
Representative cross (**A**–**C**) and horizontal (**D**–**F**) sections of cervical cord stained with antibodies against Aβ and IBA-1 in the spinal cord of 20 month-old TgCRND8 mice at 28 days following nerve ligation. (**G**): Quantitative data showed that microglia activation did not diminish Aβ plaque deposition in the ipsilateral side (ipsi) compared to the contralateral side (contra) following nerve ligation. Scale bar in (**C**), 100 µm. (**H**): Soluble and insoluble Aβ40 or Aβ42 levels in the contralateral and ipsilateral dorsal horn of the spinal cord of TgCRND8 mice at 28 days following the spinal nerve injury by ELISA assay. Scale bar in (**F**), 200 µm.

Given it is established that amyloid deposition occurs to the same extent in both sides of dorsal horn of unlesioned animals, we would like to know whether unilateral spinal nerve ligation would affect amyloid burden in the ipsilateral spinal cord compared to its contralateral side. We assessed amyloid burden in the cervical cord of lesioned mice on the 28 days after the ligation. Figure 6 showed amyloid immunoreactivity in both the ipsilateral and contralateral dorsal horn of 20-month-old animals following the spinal nerve injury. Compared with the contralateral side (without microglia activation), no differences of amyloid immunoreactivity could be observed in the ipsilateral (with microglia activation) cross (Figure 6A–C) and horizontal sections (Figure 6D–F). Quantitative analysis of amyloid burden in the cross sections indicated no statistically significant difference between the ipsilateral and contralateral sides of mice (Figure 6G). ELISA quantification of Aβ40 and Aβ42 (in picomoles per gram of wet spinal cord weight) further revealed that neither Aβ40 nor Aβ42 levels in the TBS- or formic acid-soluble fraction were altered significantly in the ipsilateral side compared with the contralateral side (Figure 6H).

### 2.5. Blockage of Spinal TREM2 Did Not Attenuate Aβ Plaques in the Spinal Cord Following Peripheral Nerve Ligation

**Figure 7:**
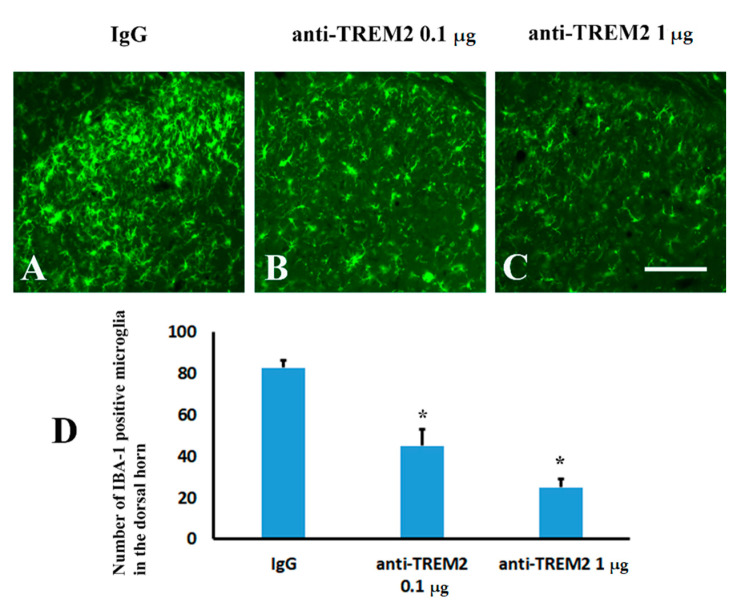
Functional blockage of TREM2 attenuated inflammatory response of spinal microglia induce by peripheral nerve ligation. (**A**): IgG. (**B**): anti-TREM2 at a dose of 0.1 µg. (**C**): anti-TREM2 at a dose of 1 µg. (**D**): Quantification of Iba1positive microglia in the ipsilateral dorsal horn following TREM2 antibody treatment in the mice following peripheral nerve injury. * *p* < 0.05 vs. IgG group. Scale bar, 150 µm.

**Figure 8:**
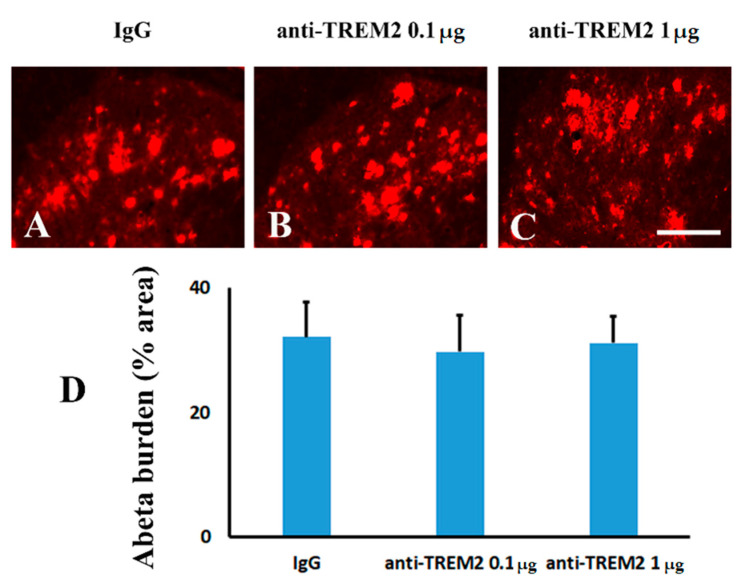
Functional blockage of spinal TREM2 did not attenuate Aβ plaques in the dorsal horn of TgCRND8 mice following peripheral nerve injury. (**A**): IgG. (**B**): anti-TREM2 at a dose of 0.1 µg. (**C**): anti-TREM2 at a dose of 1 µg. (**D**): Quantification of Aβ plaques in the ipsilateral dorsal horn following TREM2 antibody treatment in the mice following peripheral nerve injury. Scale bar, 150 µm.

In order to study the role of TREM2 in Aβ neuropathology, an anti-TREM2 neutralizing antibody was intrathecally injected immediately after peripheral nerve ligation. Consistent with previous studies [25], we detected blockage of TREM2 attenuated the activation of microglia. Either 0.1 (Figure 7B) or 1 µg (Figure 7C) of anti-TREM2 neutralizing antibody significantly altered microglial activation when compared to IgG (Figure 7A) treatment in the mice following peripheral nerve ligation (Figure 7D). These data indicated that TREM2 mediated the inflammatory response of microglia to peripheral nerve injury. However, either low (Figure 8B) or high (Figure 8C) dose of the anti-TREM2 neutralizing antibody used did not remarkably attenuated Aβ plaques in the spinal cord when compared to IgG (Figure 8A) (Figure 8D). These data illustrated the functional blockage of TREM2 did not affect Aβ plaques. 

## 3. Discussion

### 3.1. TREM2 Elevation Did Not Affect Aβ Plaque Deposition in Aged Tgcrnd8 Mice

One of the major markers of AD is the extracellular deposition of Aβ peptide in the brain parenchyma as Aβ plaques. Genetic variants may lead to not only early-onset but also late-onset of AD [12]. Late-onset of AD is a more common form of AD [12]. The genetic risk factors for late-onset AD include the well-studied apolipoprotein ε4 (APOE4) alleles and the loss-of-function variants in the TREM2 gene [12]. For example, TREM2 variants gene largely increases the risk of AD. TREM2 is exclusively expressed in microglia in the brain and has been demonstrated to modulate microglia-mediated phagocytic clearance [3,4]. TREM2 deficiency in 5XFAD mice led to increased Aβ accumulation [10,11]. However, inconsistent results were also reported. In APP/PS1 mouse model of AD, loss of TREM2 was shown to ameliorated Aβ pathology by reducing neuroinflammation [14]. In the other studies, TREM2 deficiency failed to cause any significant changes in Aβ pathology in AD mice [12,13]. The inconsistency on plaque load in TREM2-deficiency AD mice might be due to different mouse models that were studied or timing at which the analyses were done. In this study, we examined whether TREM2 elevation influence Aβ plaque burden in TgCRND8 mice [20]. To facilitate analysis of the interaction of TREM2 and Aβ plaque we took advantage of the findings that aged TgCRND8 mice develop abundant Aβ plaques in the dorsal horn of the spinal cord [16,17,18] and that spinal nerve injury induces TREM2 elevation in the dorsal horn [19]. In the current study, we found the substantial elevation of TREM2 was induced in reactive microglia after spinal nerve injury in the aged mice. However, we did not observe any significant difference in Aβ plaque deposition between the ipsilateral and contralateral sides of dorsal horn. These data may not be straightforward interpretations of TREM2 on Aβ neuropathology in AD because of the complexity of our model. The peripheral nerve injury with nerve ligation may induce complicated responses in the dorsal horn of the spinal cord. For example, similar to microglia, astrocytes are known to respond to peripheral nerve insults by extending hypertrophic processes [26]. The complexity imposed by this lesion may cloud direct interpretations of TREM2 behavior. For directly addressing the effect of TREM2 on Aβ clearance, selective blockage of TREM2 by applying anti-TREM2 neutralizing antibody was further studied in the mice following the peripheral nerve injury as in previous studies [25]. Consistent with previous studies [25], we found that functional blockage of TREM2 altered spinal microglial reaction. However, we did not find any difference in Aβ plaques between TREM2 antibody treated and vehicle treated mice. Our findings suggest that TREM2 function may not affect Aβ pathology in the aged TgCRND8 mice, suggesting the phagocytic activity by microglia in the aged TgCRND8 mice cannot be improved by TREM2 elevation. Our current findings support the findings by Jiang et al. [27]. In their study, they have also found that if TREM2 elevation has no neuroprotective effects on AD-related neuropathology and cognitive functions in APPswe/PS1dE9 mice [27]. The absence of beneficial effects by TREM2 elevation in microglia may attribute to the reduced expression of Aβ-binding receptors in the aged [27], highlighting the importance of early therapeutic interventions targeting TREM2 for AD.

**Figure 9:**
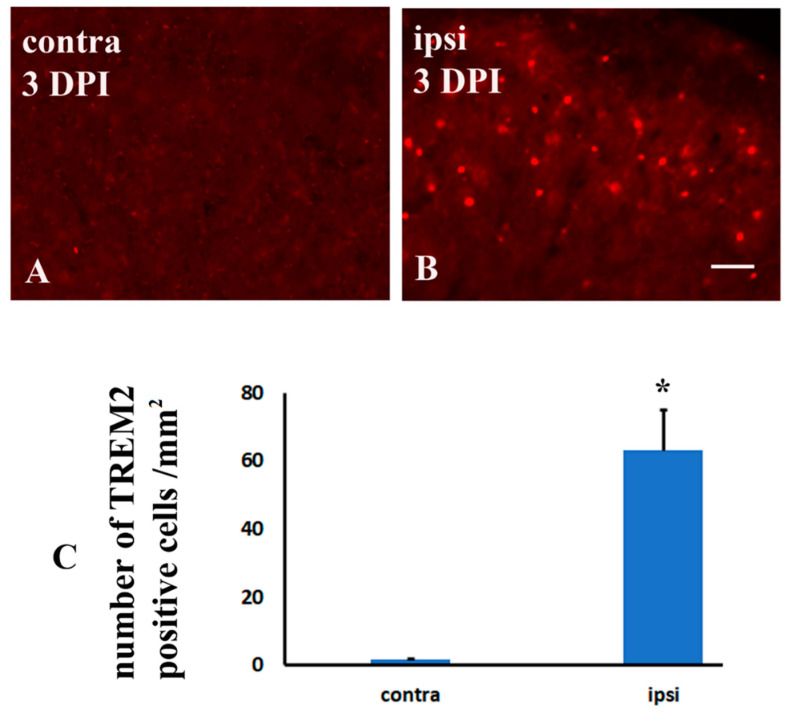
Immunohistochemistry for TREM2 in the dorsal horns of the spinal cord following nerve ligation of brachial plexus in non-TgCRND8 mice. (**A**): TREM2 expression of the contralateral side (contra) at 3 days following nerve ligation (DPI). (**B**): TREM2 expression of the ipsilateral sides (ipsi) at 3 days after nerve ligation (DPI). (**C**): Comparison in the TREM2 expression at 3 days after nerve ligation between the contralateral and ipsilateral side of the dorsal horn. * Significant differences between the two sides, *p* < 0.001. Scale bar, 50 μm.

Of note, no significant TREM2 microglia was found in the contralateral side of the spinal cord with heavy amyloid deposition, which showed the same manner with non-transgenic mice (Figure 9). Like in the aged TgCRND8 mice, we also found that TREM2 was induced in the ipsilateral side of non-transgenic mice following nerve ligation (Figure 9), suggesting that TREM2 is induced by nerve ligation rather than amyloid overexpression. Thus, Aβ itself may not induce TREM2. 

### 3.2. Microglia Activation Did Not Affect Aβ Plaque Deposition in Aged Tgcrnd8 Mice 

Reactive gliosis is one of the pathological hallmarks of AD [6,14]. As in many other neurodegenerative diseases, whether the reactive gliosis observed in AD serves to drive or delay disease progression is a controversial and complex issue with respect to Aβ pathology. On the one hand, activated microglia may exert a detrimental action to AD. For example, plaque-associated glial activation induces a self-reinforcing positive feedback loop that aggravates Aβ deposition by enhancing amyloidogenic processing of APP [28,29,30,31]. The detrimental role for microglial activation was also supported by a recent finding that microglia-derived ASC specks rapidly bind to amyloid-β and increase the formation of amyloid-β oligomers and aggregates, acting as an inflammation-driven force for amyloid-β pathology [32]. On the other hand, activated microglia could also play a beneficial role. For example, increased microgliosis through overexpression of interleukin (Il)-1β or Il6 leads to amelioration of plaque pathology [33]. Similarly, decreased microgliosis through knocking out the chemokine receptor CCR2 has been shown to exacerbate Aβ pathology in the brain of APP-transgenic mice [34]. Thus, there is still no concluding agreement regarding the roles of microglia in AD. In this study, we found that spinal nerve injury by brachial plexus ligation induced robust microglial activation in the dorsal horn, as revealed by the increased number and de-ramification of microglia. However, these reactive microglia could not affect Aβ deposition. It is possible that the failure of Aβ plaque clearance was due to the short period of microglia activation (around 4 weeks) in these mice. However, Wilcock et al. [35,36] demonstrated that microglial activation led to microglia associated removal of plaques within 24 h. This evidence indicates microglia activation for 4 weeks should be sufficient for the clearance of Aβ plaques. The absence of clearance of Aβ plaques may be due to the phagocytic deficit of microglia in aged mice [27,37].

## 4. Materials and Methods

### 4.1. Animal Model

Male TgCRND8 mice were used in the current study [16,18,20,38]. The mice expressed the Indiana (V717F) and Swedish mutations (K670N / M671L) of human APP gene on a C57BL6/J genetic background [16,18,20,38]. The colony was generated by crossing heterozygous transgenics with C57BL6/J mice. Genotyping was performed by polymerase chain reaction analysis of ear genomic DNA as described in previous studies [16,17,18]. All surgical procedures and subsequent care and treatment were endorsed by the Animal Experimentation Ethics Committee (AEEC), The Chinese University of Hong Kong.

### 4.2. Spinal Nerve Injury Model and Surgical Procedures

Male TgCRND8 mice aged 20 months were anesthetized via intraperitoneal injection of ketamine (80 mg/kg) and xylazine (8 mg/kg). The surgical procedures for spinal nerve injury were performed using the described methods in our previous studies [39,40,41]. In brief, the right brachial plexus was exposed under an operating microscope at the trunk level through an infraclavicular approach. Then the right brachial plexus was then tightly ligated. After the nerve ligation, the wound was closed with 5-0 sutures. The postoperative survival period of the mice was 1, 7, 14, and 28 day/days. 

### 4.3. Intrathecal Injection of TREM2 Neutralizing Antibody

The intrathecal injection of TREM2 neutralizing antibody was performed once daily using a 10 µL Hamilton syringe with a 30 1/2-gauge needle as described in previous studies [25] in TgCRND8 mice at the age of 20 months following peripheral nerve ligation. Briefly, the needle was inserted into the intervertebral space between the L5 and L6 level of the spinal cord. An indicator of the accuracy of each injection was a reflexive flick of the tail of mice [42]. The same volume of or recombined human IgG Fc (110-HG, R&D) was intrathecally injected as control (*n* = 4 in each group).

### 4.4. Perfusion and Tissue Processing

As described in our previous studies [16], animals were deeply anesthetized at designed points with a lethal dose of pentobarbital (50 mg/kg, i.p) and then perfused intracardially with normal saline, followed by 4% paraformaldehyde in 0.1 M phosphate- buffer (PB) (pH 7.4). Cervical enlargement was dissected out, post-fixed with 4% paraformaldehyde, and then put into 30% sucrose solution in 0.1 M PB overnight. Spinal cords were cut into 25 μm-thick cross or horizontal sections using cryostat (Thermo Fisher Scientific, Chicogo, IL, USA). The sections were then collected in wells of 24-well plates containing PB for immunohistochemical analyses.

### 4.5. Immunohistochemical Analysis

For immunofluorescence, sections were incubated with primary antibodies overnight at room temperature. Antibody information including names, vendors, and concentrations are shown in Table 1. The antibody used for TREM2 in this study is from R&D Systems (shown in Table 1) as used in previous studies [9]. For double staining of Aβ/thioflavin S, the sections were incubated with anti-Aβ antibody overnight and their corresponding Alexa 488 or 568-conjugated secondary antibody (1:800, Molecular probes, Eugene, OR, USA) for 2 h at room temperature. Each step was followed by three washes in the 0.1 M PB (pH7.4). Subsequently, the sections were stained by thioflavin S for 1 min. For staining of Aβ, TREM2, IBA-1, TREM2/IBA-1, or Aβ/IBA-1, the sections were incubated with primary antibodies overnight and their corresponding Alexa 488 or 568-conjugated secondary antibodies (1:800, Molecular probes, Eugene, OR, USA) for 2 h. The sections were then placed on gelatin-coated glass slides and coverslipped in a mounting medium (Dako, Santa Clara, CA, USA). Fluorescent images were captured under a Zeiss microscope (Zeiss, Gottingen, Germany) using a Spot digital camera (Diagnostic Instruments, Sterling Heights, MI, USA). To determine Aβ plaque burden, 10 cross sections of the spinal cord were assessed for each mouse (*n* = 6). The plaque burden was expressed as the area of Aβ divided by the total area measured, as described in our previous studies [16]. To determine microglia reaction following the spinal nerve injury, the average number of the TREM2 and IBA-1 positive microglia in the contralateral and ispilateral dorsal horn area was calculated by the researchers who were blinded for group assignment (*n* = 6).

### 4.6. Assessment of Aβ Levels

To determine the levels of soluble Aβ40 and Aβ42 peptides, human β-Amyloid Aβ40 and Aβ42 colorimetric sandwich ELISA (enzyme-linked immunosorbent assay) kits (Wako Pure Chemical Corporation, Osaka, JP) were used by the 2-step sequential extraction of Tris-buffered saline and formic acid (FA) methods as described in our previous study [17]. Briefly, contralateral or ispilateral dorsal horn of the spinal cord was homogenized in Tris-buffered saline (137 mM NaCl and 20 mM Tris, pH 7.6) supplemented with protease inhibitors (Sigma). After sonication, samples were centrifuged at 100,000× *g* at 4 °C for 1 h in order to obtain a soluble fraction for assessing soluble Aβ. The resulting pellets were resuspended in a 70% formic acid solution and centrifuged at 4 °C at 100,000× *g* for 1 h. Supernatants were collected for measuring insoluble Aβ. The formic acid extracts were neutralized with 1 M Tris (pH 11) and then diluted at least 1:2000 in ELISA incubation buffer (PBS with 0.1% bovine serum albumin, 0.05% Tween 20). Absorbances were measured at 450 nm in duplicate wells. The average of the signal from 2 wells was considered to represent the Aβ concentration for the sample (*n* = 4). 

### 4.7. Statistical Analysis 

All measurements were performed by a research assistant blinded to the group assignment. The raw data were presented as mean ± SD. Between-group comparisons were made using two-tailed unpaired Mann–Whitney U-tests. Group comparisons were performed using one-way ANOVA. All statistical analyses were performed with GraphPad Prism software (Version 7.0, San Diego, CA, USA). *p* < 0.05 was considered significant. 

## 5. Conclusions

In conclusion, the present study provided evidence that Aβ load is not affected by microglial TREM2 elevation in spinal cord dorsal horn of aged TgCRND8 mice. Our study indicates that TREM2 elevation may not have a neuroprotective effect in late stages of AD, highlighting the importance of early therapeutic interventions targeting TREM2 for AD. 

## Figures and Tables

**Table 1:** Antibodies and ELISA kits used in the experiments.

Antibodies	Species	Working Dilution	Vendor or Producer
Bam-10	Mouse IgG	1:3000	Sigma
IBA-1	Rabbit IgG	1:3000	Wako
TREM2	Sheep IgG	1:1000	R & D systems
Goat anti-rabbit 488		1:800	Molecular probes
Goat anti-mouse 568 Donkey anti-sheep 568		1:800 1:800	Molecular probes Molecular probes
thioflavin S ELISA kits for Aβ40 and Aβ42		2%	Sigma Wako

## Data Availability

The data used in the current study are available from the corresponding author on request.
